# A New Method of Soldering

**Published:** 1903-04-15

**Authors:** F. W. Stephan

**Affiliations:** Chicago, Ills.


### A NEW METHOD OF SOLDERING.

BY F. W. STEPHAN, D.D.S., CHICAGO, ILLS.

Take of filings of an easy flowing solder, and filings of the gold to be soldered or some higher fusing metal, about equal parts. Also borax and water rubbed up in a mortar or otherwise to make a creamy solution. Mix the filings with sufficinet borax solution to make a thick paste. Pack the joint to be soldered with this paste and heat till fused. Care should be taken that the entire mass is evenly heated through out, and of course all the ordinary precautions of having surfaces bright and clean, etc., must be observed.

This method is especially adopted when large spaces are to be bridged, or where it is desirable to add to a cusp or to contour or otherwise change the shape of a piece in any way. Almost any form desired may be obtained, due allowance being made for shrinkage.

The particles of high fusing metal serve as a support to retain the shape of the mass, and the low fusing solder acts as a cement when fused to unite these particles and bind them to the piece being soldered.

